# Data in the time of cholera: an assessment of global data resources for optimising surveillance, response and control

**DOI:** 10.1136/bmjgh-2025-019626

**Published:** 2025-09-11

**Authors:** Tessa Rose Cornell, Louise A Kelly-Hope

**Affiliations:** 1 Institute of Infection, Veterinary and Ecological Sciences, University of Liverpool, Liverpool, UK

**Keywords:** Cholera, Global Health

## Abstract

Cholera represents a public health threat worldwide and an indicator of poverty, inequity and lack of social development, disproportionately affecting low-income and middle-income countries. Accessible global cholera data resources are essential to support timely, data-driven disease surveillance, response and control efforts. This analysis aims to identify, collate and describe online open-access cholera resources. 31 resources associated with multilateral agencies, academic institutions and non-profit organisations were identified, encompassing dashboards (n=16/31, 51.6%), reports or bulletins (n=12/31, 38.7%) and outbreak reporting systems (n=3/31, 9.7%). The majority of resources were affiliated with the WHO (n=19/31, 61.3%). Other affiliations comprised other United Nations (UN) and multilateral agencies (n=9/31, 29.0%), a non-profit organisation (n=1/31, 3.2%) and academic institutions (n=2/31, 6.5%). Most resources had global scope (n=21/31, 67.7%), provided data to national or subnational levels (n=27/31, 87.1%) and demonstrated variable temporal resolution and reporting frequency. 11 resources affiliated with national institutions were described, reporting predominantly weekly cholera data to the subnational level. Resources comprised epidemiological reports and bulletins, infectious disease dashboards and integrated disease surveillance and response platforms. This analysis highlights cholera resources available to researchers, healthcare workers and policy-makers, which may direct disease programmes and research activities and support Global Task Force on Cholera Control roadmap 2030 targets. National resources provided detailed subnational cholera data and complemented cholera reporting by multilateral agencies. Timely review of these resources is warranted.

**SUMMARY BOX**

- Cholera is a neglected public health challenge worldwide, indicative of poverty, inequity and lack of social development.
- Existing global, regional and national cholera data resources support Global Task Force on Cholera Control roadmap 2030 targets.
- Researchers, healthcare workers and policy-makers can use resources to inform and optimise critical cholera surveillance, response and control programmes.

## Cholera: promoting data sharing and access to tackle a persistent global public health threat

 Cholera, an acute diarrhoeal infection with bacterium *Vibrio cholerae*, continues to represent a public health threat worldwide. Disproportionately affecting low-income and middle-income countries (LMICs), cholera incidence is a key indicator of economic status, social equity and development, commonly affecting communities with poor sanitation and access to clean water.^1^,^6^ Cholera outbreaks, resurgence and persistence have been associated with extreme climate (eg, floods and drought), conflict and mass displacement events.^7^,^12^ 50% mortality has been reported in untreated individuals with severe disease; thus, timely and appropriate cholera case management is essential, comprising tailored oral or intravenous rehydration therapy, designated cholera treatment facilities and staff trained on treatment protocols.^13 14^ In addition, targeted deployment of oral cholera vaccines (OCVs) may be considered.^15^

From January to December 2024, 33 WHO Member States reported a cumulative total of 807 570 cholera cases and 5863 deaths, primarily across African (n=18), Eastern Mediterranean (n=8) and South-East Asia (n=5) WHO regions.^16^ However, estimates indicate that at least 2.9 million cases and 95 000 deaths occur per year worldwide, primarily across Asia and Africa.^17^ The WHO classification of cholera events as a grade 3 emergency reflects the significant number and geographical expansion of outbreaks and the continued shortage of OCVs relative to an increasing global demand.^7 16 18^ There is an urgent need for collective action and improved understanding of the spatial-temporal distribution and key drivers of this neglected disease.

The Global Task Force on Cholera Control (GTFCC) Ending Cholera—A Global Roadmap to 2030 outlines a strategy for implementing multisectoral interventions in cholera-affected countries, with the aim of reducing cholera deaths by 90% by 2030.^17^ Interventions include: reducing cholera burden, including early detection and response to contain outbreaks; prevention, by targeting interventions in cholera ‘hotspots’; and effective coordination mechanisms for technical support, resource mobilisation and partnership.^17^ In addition, the GTFCC, with the support of the WHO, coordinates the collection and sharing of data, including on the WHO Global Cholera and Acute Watery Diarrhoea (AWD) Dashboard.^16^ As per the WHO definition, AWD is defined as ‘…three or more loose or watery (non-bloody) stools within a 24-hour period’,^19^ and includes cholera.

In line with GTFCC roadmap 2030 targets, and to advance global health equity and digital literacy, availability of and engagement with open-access cholera data resources are critical. Increased understanding of the characteristics of resources affiliated with the WHO and other organisations and institutions at global, regional and national levels will support timely, data-driven national cholera surveillance, response and control efforts by researchers, healthcare workers and policy-makers. Towards these efforts, the OpenWHO learning resource platform provides open-access modules on cholera case management and priority areas for multisectoral interventions^14^; however, equitable access to these resources should be considered (eg, in areas affected by extreme climate events or conflict with restricted internet access).

The primary objective of this analysis was to identify, collate and describe the characteristics of publicly available cholera resources, which provide data to optimise cholera surveillance, response and control efforts. This review aims to direct and optimise engagement with existing resources and to promote an underlying ethos of global cholera data sharing and access. Additional resources can be added to develop a comprehensive data repository for cholera stakeholders.

## Identification of cholera data resources

Cholera data resources were identified as follows: (1) manual web-searching of targeted websites of multilateral organisations, academic institutions and non-governmental organisations (NGOs); (2) literature review on PubMED electronic database using key search terms cholera* and data* (and synonyms) and names of WHO cholera endemic countries in 2024^16^; and (3) manual web-searching of targeted websites of national public health institutions. Selection of national institutions was informed by the WHO list of cholera endemic countries^16^ and by examination of cholera data sources referenced by identified resources (eg, national data sources used for secondary data analyses in the literature or contributing to aggregated resources affiliated with multilateral agencies and academic institutions). The search was conducted between 11 November and 22 December 2024 (and on 7 July 2025 to include two additional identified resources).

Open-access online resources containing cholera data were eligible for inclusion, comprising resources with a cholera or AWD focus and resources which provided cholera data but reported more broadly on infectious diseases or public health emergencies. Data resources affiliated with multilateral agencies, academic or research institutions, non-profit or NGOs and government were examined. On the literature review, described cholera resources which had been utilised for secondary data analyses were included, but datasets generated from primary cholera research were excluded. Resource types included (but were not limited to) dashboards, bulletins or reports and outbreak reporting systems. No restrictions were placed on resource geographical scope, geographical or temporal resolution of resource data or the availability of data for 2024 (or 2025 for additional identified resources).

The search was non-exhaustive and a timely review is warranted to provide an up-to-date repository. Furthermore, the search strategy was partially informed by the WHO list of cholera endemic countries,^16^ representing a selection bias towards resources from these countries.

Resources were searched manually for the data items described in table 1. Data items were categorised into resource affiliations, resource type (including frequency of resource updates and availability of current data), resource scope (disease or public health scope and geographical scope), cholera data variables provided by resources and geographical and temporal resolutions. Data were extracted into a Microsoft Excel file for descriptive data analysis.

**Table 1:** Characteristics of cholera resources

Data item	Detail/examples	Categories (if applicable)
Resource affiliations		
Primary affiliation		Multilateral agency, academic/research institution, non-profit organisation/non-governmental organisation, government
Primary data source(s)	Source of primary data (eg, national public health institutions)	–
Funding source(s)	Source(s) of resource funding	–
Resource type		
Resource category		Dashboard*, bulletin/report, outbreak reporting system
Frequency of reports or updates	Frequency that resource adds or disseminates new data or reports	Weekly, bimonthly, monthly, annual, no set interval/24/7
Availability of 2024 data	Data from 2024 provided by resource	Yes, No
Resource scope		
Disease or Public Health scope	Disease or public health scope of resource	Cholera, cholera and acute watery diarrhoea, infectious diseases, public health emergencies/threats†, crises/disasters‡
Geographical scope	Geographical area described by resource	Global, regional, national
Resource data variables and resolution		
Data variables	Cholera data variables provided by resource as per the most recent report or update (eg, case number, death number, case fatality rate (CFR), public health interventions, age group and sex distributions)	–
Geographical resolution of data	Smallest geographical level described by cholera data	Regional, national, subnational
Temporal resolution of data	Shortest time period described by cholera data (eg, case number per week)	Weekly, monthly, annual, no set interval

* Dashboard=Online resource summarising related data sets.

† Public health emergency/threat: Definition as per IHR (2005) definition of Public Health Emergency of International Concern.^26^

‡ Disaster=Unforeseen and often sudden events that cause significant damage, destruction and human suffering.^22^

IHR, International Health Regulations.

## Compilation and characteristics of cholera resources

### Resources affiliated with multilateral agencies, NGOs and academic institutions

#### Resource identification and affiliations

In total, 31 resources were identified, providing global or regional level data on cholera surveillance, response and control. Resource characteristics are summarised in table 2. Resource affiliations comprised WHO and WHO Regional Offices (n=19/31, 61.3%), other UN or multilateral agencies (n=9/31, 29.0%), academic institutions (n=2/31) and a non-profit organisation (n=1/31). Full references for identified resources are provided in online supplemental appendix A and correspond to reference codes (a–z) and (aa–ee) in table 2.

**Table 2:** Characteristics of open-access cholera data resources, affiliated with multilateral agencies, non-governmental organisations and academic institutions

	Resource affiliation								Summary, N=31
	WHO, N=19 (61.3%) (a–s)	ECDC, N=3 (9.7%) (t–v)	UNICEF, N=4 (12.9%) (w–z)	UNOCHA, N=1 (3.2%) (aa)	UNHCR, N=1 (3.2%) (bb)	ISID, N*=*1 (3.2%) (cc)	CRED, N*=*1 (3.2%) (dd)	Johns Hopkins University (3.2%) (ee)	
Affiliation category	Multi-lateral agency					Non-profit organisation	Academic institution		
Frequency, n (%)									
Resource type									
Resource category*									
Dashboard†	11 (57.9)	1 (33.3)	3 (75.0)	–	–	–	–	1 (100.0)	16 (51.6)
Report/bulletin	8 (42.1)	2 (66.7)	1 (25.0)	–	1 (100.0)	–	–	–	12 (38.7)
Outbreak reporting system	–	–	–	1 (100.0)	–	1 (100.0)	1 (100.0)	–	3 (9.7)
Frequency of reports or updates									
Weekly	3 (15.8)	1 (33.3)	–	–	–	–	–	–	4 (12.9)
Twice monthly	2 (10.5)	–	2 (50.0)	–	–	–	–	–	4 (12.9)
Monthly	2 (10.5)	1 (33.3)	–	–	1 (100.0)	–	–	–	4 (12.9)
Annual	–	1 (33.3)	–	–	–	–	–	–	1 (3.2)
No set interval/24/7	2 (10.5)	–	1 (25.0)	1 (100.0)	–	1 (100.0)	1 (100.0)	1 (100.0)	7 (22.6)
Unknown	10 (52.6)	–	1 (25.0)‡	–	–	–	–	–	11 (35.5)
Availability of 2024 (or 2025) Data									
Yes	7 (36.8)	2 (66.7)	2 (50.0)	1 (100.0)	1 (100.0)	1 (100.0)	1 (100.0)	1 (100.0)	16 (51.6)
No	12 (63.2)	1 (33.3)	1 (25.0)	–	–	–	–	–	14 (45.2)
Unknown	–	–	1 (25.0)§	–	–	–	–	–	1 (3.2)
Resource scope									
Disease or public health scope									
Cholera	12 (63.2)	2 (66.7)	3 (75.0)	–	–	–	–	1 (100.0)	18 (58.1)
Cholera/AWD	2 (10.5)	–	1 (25.0)	–	–	–	–	–	3 (9.7)
Infectious disease (general)	4 (21.1)	–	–	–	–	1 (100.0)	–	–	5 (16.1)
Public health emergency/threat¶	1 (5.3)	1 (33.3)	–	–	–	–	–	–	2 (6.5)
Crisis/disaster§	–	–	–	1 (100.0)	–	–	1 (100.0)	–	2 (6.5)
Refugee emergency	–	–	–	–	1 (100.0)	–	–	–	1 (3.2)
Geographical scope									
Global	14 (73.7)	2 (66.7)	–	1 (100.0)	1 (100.0)	1 (100.0)	1 (100.0)	1 (100.0)	21 (67.7)
Regional	5 (26.3)	1 (33.3)	3 (75.0)	–	–	–	–	–	9 (29.0)
National	–	–	1 (25.0)	–	–	–	–	–	1 (3.2)
Resource data resolution									
Geographical resolution of data									
Regional	3 (15.8)	–	–	–	–	–	–	–	3 (9.7)
National	11 (57.9)	3 (100.0)	–	–	–	–	–	1 (100.0)	15 (48.4)
Subnational	5 (26.3)	–	3 (75.0)	1 (100.0)	1 (100.0)	1 (100.0)	1 (100.0)	–	12 (38.7)
Not specified/unknown	–	–	1 (25.0)§	–	–	–	–	–	1 (3.2)
Temporal resolution of data									
Weekly	6 (31.6)	1 (33.3)	1 (25.0)	–	–	–	–	–	8 (25.8)
Monthly	–	1 (33.3)	1 (25.0)	–	–	–	–	–	2 (6.5)
Annual	10 (52.6)	1 (33.3)	–	–	–	–	–	–	11 (35.5)
No set interval	3 (15.8)	–	–	1 (100.0)	1 (100.0)	1 (100.0)	1 (100.0)	1 (100.0)	8 (25.8)
Not specified/unknown	–	–	2 (50.0)§	–	–	–	–	–	2 (6.5)

Full references for identified resources (codes (a–z) and (aa–ee)) are provided in online supplemental appendix A.

* Categorised as primary resource type (eg, a dashboard containing reports/bulletins was categorised as a dashboard).

† Dashboard=online resource summarising related data sets.

‡ Dashboard (n=1) under construction (access date 12 November 2024).

§ Disaster=Unforeseen and often sudden events that cause significant damage, destruction and human suffering.^22^

¶ Public Health Emergency/ Threat: Definition as per IHR (2005) definition of Public Health Emergency of International Concern.^26^

AWD, acute watery diarrhoea; CRED, Centre for Research on the Epidemiology of Disasters, University of Louvain; ECDC, European Centre for Disease Prevention and Control; ISID, International Society for Infectious Diseases; UNHCR, United Nations High Commissioner for Refugees; UNICEF, United Nations Children’s Fund; UNOCHA, United Nations Office for the Coordination of Humanitarian Affairs.

Resources contained aggregated cholera datasets and descriptive statistics. Primary sources of data included national government institutes of WHO Member States and European Union/European Economic Area (EU/EEA) Member Countries, NGOs and humanitarian agencies, research institutions, the media and the public. Details of resource funding sources are provided in online supplemental appendix B.

#### Resource type

As summarised by table 2, resources comprised predominately dashboards (n=16/31, 51.6%) and reports or bulletins (n=12/31, 38.7%), which were primarily aggregated datasets collated from multiple data sources. ReliefWeb ^20^, ProMED^21^ and EM-DAT^22^ online outbreak reporting systems were identified, affiliated with a UN agency (UNOCHA), a non-profit organisation and an academic institution, respectively. Resources disseminated new data or reports at variable set frequencies or no set intervals, as demonstrated by the outbreak reporting systems, Johns Hopkins University cholera data repository^23^, and WHO-affiliated emergency situation reports^24^ and Disease Outbreak News.^25^ Only 51.6% (n=16/31) of resources provided cholera data for 2024 (or 2025, n=2 additional identified resources).

#### Resource scope

18 (58.1%) data resources collected cholera data exclusively, of which 66.7% (n=12/18) were WHO-affiliated, summarised by table 2. The remaining resources collected data on the following: cholera and AWD (n=3/31, 9.7%); infectious diseases (n=5/31, 16.1%); public health emergencies or threats (n=2/31, 6.5%), as defined by the IHR (2005)^26^; crises or disasters (n=2/31, 6.5%), as defined by EM-DAT reporting system^22^; and refugee emergencies (n=1/31).^27^ Resources provided global (n=21/31, 67.7%), regional (n=9/31, 29.0%) and national data (n=1/31).

#### Resource data variables and resolution

The majority of data resources provided cholera case numbers (n=29/31, 93.5%), deaths (n=26/31, 83.9%) and case fatality rate (CFR; n=15/31, 48.4%). Details of cholera data variables are summarised for each resource in online supplemental appendix B. The majority of resources provided cholera data to national (n=15/31, 48.4%) and subnational (n=12/31, 38.7%) levels, summarised by table 2. Resources demonstrated variable temporal data resolution, with the majority providing cholera statistics (eg, case numbers or deaths) per week or annually.

### Resources associated with national institutions

#### Resource identification and affiliations

11 resources providing cholera data were described, affiliated with government ministries and national institutions associated with health, public health and epidemiology and disease control, summarised by table 3. Full references for identified resources are provided in online supplemental appendix A and correspond to reference codes (ff–pp) in table 3.

**Table 3:** Summary of national cholera data resources and associated characteristics

Resource	Country	Data source(s)	Disease or public health scope	Geographical resolution of data	Temporal resolution of data	Frequency of reports or updates	Reference
Cholera cases reported in 2024^16^							
WHO AFRO							
NCDC National Disease Outbreak Dashboard 2006–2023	Nigeria	Nigeria Centre for Disease Control and Prevention (NCDC)	Infectious diseases (general)	Subnational	Weekly	Unknown	(ff)
NCDC Cholera situation report (monthly epidemiological report)	Nigeria		Cholera	Subnational	Weekly	Monthly	(gg)
Ministry of Health The Weekly Epidemiological Bulletin	Uganda	Ministry of Health, Integrated Epidemiology, Surveillance and Public Health Emergencies Department	Public health emergencies	Subnational	Weekly	Weekly	(hh)
Zambia Cholera Outbreak Situation Report	Zambia	Ministry of Health	Cholera	Subnational	Weekly	No set interval	(ii)
WHO EMRO							
IDSR Weekly Bulletin	Pakistan	Center of Disease Control, National Institute of Health	Infectious diseases (general), Dog bites	Subnational	Weekly	Weekly	(jj)
Electronic Integrated Disease Early Warning System–Epidemiological Situation of diseases in free areas in Yemen, 2021–2024 (W1–W36)	Yemen	Ministry of Public Health and Population	Infectious diseases (general)	Subnational	Weekly	Weekly	(kk)
South Sudan: Weekly Integrated Disease Surveillance and Response bulletin	South Sudan	Ministry of Health (National surveillance officers)	Infectious diseases (general)(priority diseases, events and conditions)	Subnational	Weekly	Weekly	(ll)
e-IDSR Weekly Epidemiological Bulletin	Somalia	National Public Health Reference Laboratory	Infectious diseases (general)	Subnational	Weekly	Weekly	(mm)
WHO SEAR							
EWARS Weekly Bulletin	Nepal	Department of Health Services Epidemiology and Disease Control Division	Infectious diseases (general)	Subnational	Weekly	Weekly	(nn)
Situation Update on Cholera	Nepal		Cholera	Subnational	Weekly	No set interval	(oo)
No cholera cases reported in 2024^16^							
US CDC	USA	National public health agencies (state, local, territorial)	Cholera/ AWD	Subnational	Annual	Annual	(pp)

Full references for identified resources (codes (ff–pp)) are provided in online supplemental appendix A.

AWD, acute watery diarrhoea; CDC, Centers for Disease Control and Prevention; EWARS, Early Warning and Reporting System; IDSR, Integrated Disease Surveillance and Response; NCDC, Nigeria Centre for Disease Control and Prevention; WHO AFRO, WHO Africa Regional Office; WHO EMRO, WHO Eastern Mediterranean Regional Office; WHO SEAR, WHO South-East Asia Regional Office.

#### Resource type

**Figure 1:**
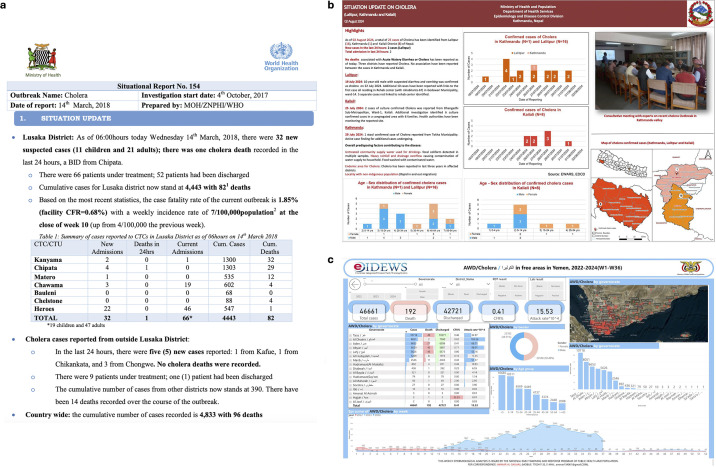
Examples of open-access cholera data resources associated with government institutions in (**a**) Zambia (Ministry of Health and WHO, https://www.afro.who.int/sites/default/files/2018-03/Zambia_Sitrep_Cholera-14March2018.pdf, access date 28/07/25)^32^, (**b**) Nepal (Ministry of Health and Population, https://edcd.gov.np/uploads/news/pdf/66acc33a92d18.pdf, access date 28/07/25)^41^ and (**c**) Yemen (Ministry of Public Health and Population, https://app.powerbi.com/view?r=eyJrIjoiNjEzY2NkNzItY2YxMC00Mjk4LTlmOGItYzgzYmUxMjY2OWRjIiwidCI6IjRhYzMyMjllLTVlMzctNDcyNS1hY2I1LWU2MjhjNjIzNWE5ZiIsImMiOjl9&pageName=ReportSectionaa3efb8cb5fd4cba8386, access date 28/07/25)^42^. AWD, acute watery diarrhoea; CFR, case fatality rate; CTU, cholera treatment unit; EDCD, Epidemiology and Disease Control Division; EWARS, Early Warning and Reporting System.

National resources comprised epidemiological reports and bulletins, infectious disease dashboards and integrated disease surveillance and response (IDSR) platforms (table 3, figure 1). Resources disseminated new data or reports at variable set frequencies, with weekly publication of six resources, comprising national epidemiological bulletins (n=5/11, 45.5%) and one disease dashboard. The majority of identified resources (n=8/11, 72.7%) provided cholera data for 2024.

#### Resource scope

Identified resources, demonstrating national scope, provided data on infectious diseases (n=6/11, 45.5%), cholera exclusively (n=2/11), cholera and AWD (n=1/11), cholera and vibriosis (n=1/11) and public health emergencies (n=1/11; table 3).

#### Resource data variables and resolution

Cholera data variables provided by national resources included case number (n=11/11), deaths (n=7/11, 63.6%) and CFR (n=4/11, 36.4%). Variable additional data were provided, which related to the following: case demographics (age group and sex distributions); case numbers admitted, under treatment, discharged or referred; case confirmation (by culture, rapid diagnostic tests or microscopy); national or subnational outbreak response activities and public health interventions; and *V. cholerae* serogroup and serotype. Details of cholera data variables are summarised for each resource in online supplemental appendix B.

All national resources provided cholera data to the subnational level. 90.9% (n=10/11) resources provided weekly case numbers.

## Conclusions

This analysis has identified cholera resources affiliated with global, regional and national public health agencies, organisations and institutions, which can support worldwide cholera disease surveillance, response and control efforts. These efforts align with targets of the GTFCC roadmap 2030,^17^ regional frameworks (eg, the 2018 WHO-Africa Regional Office cholera prevention and control framework)^28^, and national cholera plans.^29^ In line with Axis 2 of the GTFCC roadmap, the availability of open-access morbidity and mortality data enables the identification of vulnerable populations and high-risk areas which inform cholera control efforts, including the design and implementation of multisectoral interventions. These data contribute to the development of national cholera control programmes. Furthermore, in line with Axis 3 of the GTFCC roadmap, cholera data sharing among national governments and global stakeholders facilitates effective coordination mechanisms for cholera control and response. Thus, this review highlights current cholera data resources which support global cholera control efforts and further research opportunities, as well as current resource limitations.

This analysis is non-exhaustive; thus, timely review of identified resources and the addition of unidentified or new resources are warranted, to develop a comprehensive and up-to-date repository for programme managers, researchers, front-line healthcare workers and policy-makers. This would complement existing open-access cholera repositories which present aggregated data from multiple sources, such as the Johns Hopkins University-affiliated repository for global cholera reports and surveillance data.^23^ Cholera and infectious disease dashboards, reports and bulletins, affiliated with government health ministries and national institutions have been highlighted, which provide detailed epidemiological and public health data at the subnational level to explore spatial temporal dynamics and key drivers of cholera outbreaks. This is exemplified in the literature where data from national resources have been examined to explore cholera epidemiology in Zambia, Uganda and Nigeria, including identification of cholera hotspots^30 31^ and associations with socioeconomic factors^3^ and conflict events.^8^ Government resources complement those affiliated with the WHO and other multilateral agencies, which present aggregated data and descriptive summary statistics. In addition, WHO Regional Offices provide variable levels of technical support to ministries and national public health institutions to produce country-level epidemiological disease bulletins and situation reports.^32^,^35^ This analysis has examined those resources which have been collated or coauthored primarily by national bodies, with WHO support (eg, the ministerial or institutional logo is visible).^32 35^ However, WHO Regional Office websites can be referred to for additional country-level resources which have been compiled by WHO personnel.^36 37^

Limitations related to the representativeness of the resources presented included collation of only cholera statistics presented by the most recent reports or bulletins for each resource. Furthermore, the format of cholera resources was not standardised (eg, in relation to cholera statistics or graphics presented), and open-access downloadable raw data files associated with the descriptive statistics presented were not available. More detailed comparison on and access to primary datasets at the national level are warranted, which may inform improved standardisation of cholera data collection and surveillance procedures across variable contexts. Furthermore, clearer signposting to primary data sources (eg, by aggregated data resources affiliated with multilateral agencies) will promote equitable access to and engagement with national resources.

Intersectoral communication and collaboration between national public health bodies, policy-makers and academic institutions are warranted,^17^ to increase engagement with open-access cholera datasets. Furthermore, comparison of existing resources would support the coordination (and avoid the duplication) of data collection efforts, to optimise existing resources and to identify and address evidence gaps on cholera epidemiology in underrepresented geographical regions.

The GTFCC website provides a central repository of cholera resources to support and promote the development and implementation of national cholera plans in affected countries.^38^ These include an online database of global cholera research activities related to the following GTFCC roadmap 2030 pillars: case management; community engagement; epidemiology surveillance; laboratory surveillance; vaccines and water, sanitation and hygiene.^39^ This platform could also be used to promote the resources identified in this analysis, to advance equitable access to and engagement with existing open-access cholera data. In addition, the recent launch of a new GTFCC website, with templates for cholera reporting and national cholera plans and a GTFCC OCV Dashboard, may provide additional analytical opportunities.^38^

The acute upsurge of the current seventh cholera pandemic since mid-2021, characterised by the severity, spread and concurrence of multiple outbreaks, highlights the need for continued commitment to achieving cholera reduction and elimination milestones.^40^ Cholera data collection and dissemination to policy-makers, in addition to strengthened government commitments and ownership, are essential to support these global cholera response and control efforts.

## Supplementary material

10.1136/bmjgh-2025-019626 online supplemental file 1

10.1136/bmjgh-2025-019626 online supplemental file 2

## Data Availability

All data relevant to the study are included in the article or uploaded as supplementary information.
